# PRP: pathogenic risk prediction for rare nonsynonymous single nucleotide variants

**DOI:** 10.1007/s00439-025-02751-z

**Published:** 2025-05-29

**Authors:** Jee Yeon Heo, Ju Han Kim

**Affiliations:** 1 Division of Biomedical Informatics, Seoul National University Biomedical Informatics (SNUBI), Seoul National University College of Medicine, Seoul, Korea; 2 Department of Neuropsychiatry, Seoul National University Hospital, Seoul, 03080 Korea

## Abstract

Reliable prediction of pathogenic variants plays a crucial role in personalized medicine, which aims to provide accurate diagnosis and individualized treatment using genomic medicine. This study introduces PRP, a pathogenic risk prediction for rare nonsynonymous single nucleotide variants (nsSNVs), including missense, start_lost, stop_gained, and stop_lost variants. PRP was designed to provide robust performance and interpretable predictions using thirty-four features across four categories: frequency, conservation score, substitution metrics, and gene intolerance. Five machine-learning (ML) algorithms were compared to select the optimal model. Hyperparameter optimization was conducted using Optuna, and feature importance was analyzed using Shapley Additive exPlanations (SHAP). PRP used ClinVar data for training and evaluated performance using three independent test datasets and compared it with that of twenty other prediction tools. PRP consistently outperformed state-of-the-art tools across all eight performance metrics: AUC, AUPRC, Accuracy, F1-score, MCC, Precision, Recall, and Specificity. In addition to achieving high sensitivity and high specificity without overestimating the number of pathogenic variants, PRP demonstrates robustness in predicting rare variants. The datasets and codes used for training and testing PRP, along with pre-computed scores, are available at https://github.com/DNAvigation/PRP.

**Supplementary Information:**

The online version contains supplementary material available at 10.1007/s00439-025-02751-z.

## Introduction

Comprehensive assessment of genetic variation using exome or genome sequencing to identify disease-causing variants is becoming increasingly routine in clinical genetics. Among these variants, single nucleotide variants (SNVs) are the most prevalent, accounting for approximately 0.1% of the human genome and translating to approximately 3.5 million per individual (Lin et al. 2023). These SNVs play a crucial role in the genetic diversity of human populations by influencing traits such as disease susceptibility and drug response (Sun and Yu 2019). However, the ability to interpret the vast number of genetic variants remains limited, presenting a significant challenge in effectively utilizing this data (Marian 2020).

Nonsynonymous single nucleotide variants (nsSNVs), which directly affect amino acid substitution, account for more than half of the 20,000 SNVs in the human exome (Lin et al. 2023; Shihab et al. 2014). These nsSNVs can lead to severe diseases by significantly altering protein structure or function. Therefore, distinguishing pathogenic from benign variants is critical for advancing personalized medicine. And approximately 85% of these nsSNVs have alternative allele frequencies (AFs) below 0.5%, with roughly 100–400 rare variants identified per sequenced individual (Genomes Project et al. 2012; Tennessen et al. 2012).

Experimental validation of these nsSNVs is impractical for large-scale studies because it is costly and time-consuming (Livesey and Marsh 2022). To overcome these limitations, numerous computational tools have been developed to predict the potential impact of nsSNVs. These tools utilize a variety of variant properties, including sequence homology (Reva et al. 2011), evolutionary conservation (Cooper et al. 2010), allele frequency (AF) (Alirezaie et al. 2018), physiochemical and biochemical properties of amino acids (Lu et al. 2015), protein structure (Schmidt et al. 2023), and various other prediction scores (Rentzsch et al. 2019). A wide range of algorithms have been employed in these tools, from traditional machine learning algorithms such as random forest (RF) (Carter et al. 2013), support vector machine (SVM) (Lu et al. 2015), and logistic regression (LR) (Lu et al. 2015), to the latest advancements in deep learning, including deep neural networks (DNN) (Quang et al. 2015), recurrent neural networks (RNN) (Li et al. 2022), and deep residual neural networks (ResNet) (Qi et al. 2021).

However, these tools have several limitations. Most prediction tools tend to overestimate the number of pathogenic variants, resulting in high sensitivity, low specificity, and conflicting results (Bu et al. 2022; Gunning et al. 2021; Ioannidis et al. 2016; Li et al. 2014; Zeng et al. 2024). Moreover, they primarily focus on missense variants, neglecting other variant types in coding regions, such as start_lost, stop_gained, and stop_lost. Additionally, many ensemble-based tools rely on multiple other prediction scores as features to boost performance, which can lead to issues when those scores are missing, leaving many variants unclassified (Dong et al. 2015; Li et al. 2014, 2018). Furthermore, their predictive performance for rare benign variants is notably poorer compared to that for common variants (Ioannidis et al. 2016).

To address these limitations, this study introduces PRP, a novel Pathogenic Risk Prediction method for rare nsSNVs, designed to provide robust performance and interpretable predictions utilizing new features and advanced algorithms, without relying on other prediction scores. PRP integrated features from four categories such as frequency, conservation score, substitution metrics, and gene intolerance. Neighbor Preference Frequency (NPF) was used as a feature, leveraging the fact that amino acids have different preferences for neighbors. This indicates that amino acids with similar neighbor preferences tend to replace each other more frequently than those with different neighbor preferences (Xia and Xie 2002). It was also examined whether human-specific substitution metrics are valuable as a feature in the prediction of pathogenic variants. Considering that the distribution of variants is not random across the genome and that some regions exhibit strong selection against them (Karczewski et al. 2020), features at the codon, domain, and gene levels were used.

To develop a superior model, three tree-based gradient-boosting algorithms and two deep-learning-based algorithms were applied and compared. Hyperparameter optimization was performed using Optuna (Akiba et al. 2019), and Shapley Additive exPlanations (SHAP) (Lundberg 2017) were applied to investigate the feature influence.

PRP provides more accurate performance and interpretable models for pathogenic variants than other prediction tools, thereby facilitating the identification of pathogenic variants and enhancing the utility of sequencing data in clinical genomics.

## Materials and methods

**Fig. 1:**
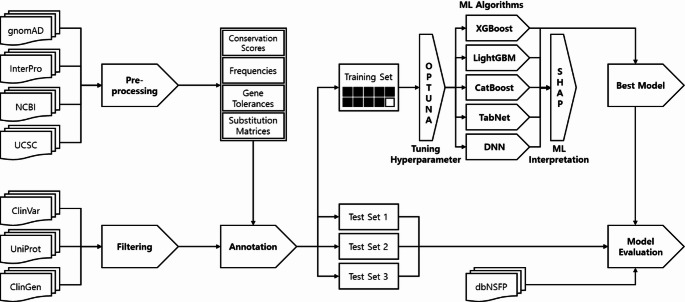
The flowchart of the PRP. Data preparation and model development and evaluation

The methodology is summarized in Fig. 1. Dataset preparation, which included filtering, preprocessing, and annotation, was performed using an in-house customized pipeline written in Perl on Linux. The model development and evaluation were conducted using Python on the Google Colab platform. Canonical transcripts were used to annotate variants under the GRCh38 reference assembly.

### Training dataset

The training dataset was sourced from the ClinVar (Landrum et al. 2018) database (clinvar_20230826.vcf.gz), which comprises clinically observed genetic variants. ClinVar includes a wide range of variant types located not only in coding regions but also in non-coding regions, with well-curated classifications distinguishing pathogenic from benign variants. The variants were filtered based on the following criteria. First, nsSNVs, including missense, start_lost, stop_gained, and stop_lost variants in coding regions, were selected. Second, nsSNVs with the clinical significance classified as pathogenic, likely pathogenic, or pathogenic/likely pathogenic were labeled as true positives (TPs), while those classified as benign, likely benign, or benign/likely benign were labeled as true negatives (TNs). Third, to reduce false positives in the curated data, nsSNVs with the review status of practice guidelines, reviewed by an expert panel, or criteria provided multiple submitters, no conflicts were retained. After filtering, 47,883 nsSNVs remained, consisting of 26,383 TPs and 21,500 TNs. To improve the classification of rare variants, 3,000 rare TNs were randomly selected and added. These variants were chosen based on the same criteria up to the second one described above, with the review status of criteria provided by a single submitter and an AF of less than 3e-4. The AF of 3e-4 corresponds to the first quartile of AFs among the 21,500 TNs. Since the number of TNs with AFs below this value was substantially lower than that of TPs, 3,000 additional TN variants were selected to balance the two classes within this low-AF range. Ultimately, the training dataset comprised 50,883 nsSNVs, including 26,383 TPs and 24,500 TNs, originating from 4,596 genes. The variant types included in the training dataset consisted of 37,803 (74.29%) missense, 335 (0.66%) start_lost, 12,720 (25%) stop_gained, and 25 (0.05%) stop_lost variants (Table S1), and the dataset was used for model development.

### Test dataset

Three distinct test datasets were compiled to assess the performance and generalizability of the model. To avoid Type 1 circularity, where the model may overestimate its performance by using the same or highly similar data for both training and evaluation, variants in the test datasets that overlapped with the training dataset were excluded (Grimm et al. 2015). In addition, overlapping variants among the three test datasets were removed to ensure independence between datasets. Variants with conflicting clinical interpretations across datasets were also excluded to maintain consistency and reduce potential bias. Furthermore, to avoid potential overlap with the training datasets of other prediction tools, which were published by 2022 and constructed using data available before that year, variants that were newly registered in 2022 or later were selected as the test dataset.

Test Dataset 1 was obtained from the latest ClinVar data (clinvar_20231230.vcf.gz). by applying the same filtering criteria used for the training dataset, and nsSNVs overlapping with the training dataset were removed. This resulted in 4,920 nsSNVs, including 2,841 TPs and 2,079 TNs from 1,813 genes, being used. Test Dataset 2 was sourced from Humsavar (release 2022_05) in UniProt (Mottaz et al. 2010; The UniProt 2017), which consists solely of missense variants curated from the literature. Variants classified as LP/P (likely pathogenic or pathogenic) were retained as TPs, while those classified as LB/B (likely benign or benign) were retained as TNs. Variants overlapping with the training dataset and Test Dataset 1 were removed, and those registered before 2022 were excluded, yielding 13,127 nsSNVs, comprising 6,840 TPs and 6,287 TNs from 4,986 genes. Test Dataset 3 was obtained from the Clinical Genome Resource (ClinGen) (Rehm et al. 2015), which provides a centralized database for the evidence-based classification of variants, supporting precision medicine and clinical decision-making. ClinGen includes various variant types located in coding regions, with well-curated classifications distinguishing pathogenic from benign variants. First, nsSNVs were selected. Second, nsSNVs with the assertion categorized as pathogenic or likely pathogenic were retained as TPs, while those categorized as benign or likely benign were retained as TNs. Third, to ensure the independence of this dataset, nsSNVs that overlapped with the training dataset and other test datasets were removed, and only nsSNVs with the approval date after 2022 were included. This dataset consisted of 282 nsSNVs, comprising 239 TPs and 43 TNs from 37 genes. The number and proportion of variant types for each test dataset were listed in Table S1.

### Dataset annotation

Thirty-four features from four categories were used to predict pathogenic variants, as listed in Supplementary Table S2.

First, frequencies related to allele frequency (AF), codon usage frequency (CF) of the codon containing the variant, and neighbor preference frequency (NPF) of amino acids adjacent to the variant were employed. AFs were obtained from gnomAD (Karczewski et al. 2020), covering all the exomes in versions 2 and 4. CF, which represents codon usage frequency in humans, was obtained from the Codon Statistics Database (Subramanian et al. 2022), which provides codon usage statistics for all species with reference or representative genomes in RefSeq. NPF was calculated using a human protein reference sequence from NCBI, applying the formula below (Xia and Xie 2002):

$$\:f\left({A}_{ijk}\right)=\frac{\sum\:_{i=1}^{20}\sum\:_{k=1}^{20}n\left({A}_{ijk}\right)}{n\left({A}_{j}\right)}$$

where $\:n\left({A}_{j}\right)$ is the number of amino acids $\:j$ in the protein reference sequence and $\:n\left({A}_{ijk}\right)\:$ is the number when the center amino acid is $\:j$, the forward amino acid is $\:i$ and the backward amino acid is $\:k$. And $\:{A}_{ijk}$ is amino acid triplets. Amino acids have distinct neighbor preferences, which influence their placement in different secondary structures. It is known that amino acids with similar neighbor preferences tend to substitute for one another more frequently than those with different preferences (Xia and Xie 2002). A higher NFP indicates similar neighbor preferences, while a lower NFP suggests differing preferences.

Second, conservation scores, including PhyloP (100way, 470way) (Pollard et al. 2010), PhastCons (100way, 470way) (Siepel et al. 2005), and multiz100way (exonNuc, exonAA), were obtained from UCSC (Kent et al. 2002). These scores were calculated based on the multiple sequence alignments of various species. PhyloP and PhastCons were utilized not only at the allele level but also at the codon, domain, and gene levels, where scores were averaged across these regions. The positions of the domains were obtained using the InterPro (Blum et al. 2021) API, and the gene structure was acquired from GenBank’s GFF file (Benson et al. 2013). The nucleotide and amino acid frequencies of the multiz100way were calculated using the formula below (Capriotti and Fariselli 2022):

$$\:f\left({x}_{i}\right)=\frac{n\left({x}_{i}\right)}{{\sum\:}_{i=1}^{i=k}n\left({x}_{i}\right)}$$

where $\:n\left({x}_{i}\right)$ is the number of the nucleotide or amino acid $\:{x}_{i}$ in the sequence alignment and $\:k$ is equal to 5 (including the generic nucleotide N) and 20 for DNA and protein sequences, respectively.

Third, substitution metrics included BLOSUM62 (Henikoff and Henikoff 1992), PAM250 (Dayhoff et al. 1978), and Grantham (Grantham 1974), as well as codon substitution (codonST) and amino acid substitution (aaST) (Shauli et al. 2021). BLOSUM62, PAM250, and Grantham are cross-species substitution metrics used to score the alignments between protein sequences. BLOSUM62 and PAM250 primarily focus on evolutionary distances, whereas Grantham assessed physiochemical differences based on the volume, polarity, and chemical properties of the side chains between amino acids. codonST and aaST are human-specific substitution metrics calculated using the ExAC database (Lek et al. 2016) and obtained from Tair et al (Shauli et al. 2021).

Lastly, gene intolerance scores, including pLI (probability of being loss-of-function intolerant), pRec (probability of being recessive), and pNull, were obtained using gnomAD v4. pLI, pNull, and pRec are scores related to gene constraints that measure the intolerance of a gene to loss-of-function (LoF) mutations.

Spearman rank correlation coefficients were calculated to assess the relationships between the features. To enhance model performance and ensure stability, the features were normalized, and missing values were imputed. A summary of the features and their corresponding missing values is provided in Table S2.

### Model development and interpretability

Five ML algorithms were applied and compared for modeling: tree-based gradient boosting algorithms XGBoost (eXtreme Gradient Boosting) (Chen and Guestrin 2016), LightGBM (Light Gradient Boosting Machine)(Ke et al. 2017), CatBoost (Prokhorenkova et al. 2018), as well as the deep learning based TabNet (Arik and Pfister 2021), and DNN (Deep Neural Network) (Montavon et al. 2018).

To fine-tune the models with more generalizability and prevent overfitting, the hyperparameter values of each algorithm were optimized using ten-fold cross-validation (CV) over the training dataset. Hyperparameter optimization was conducted using the Bayesian optimization library, called Optuna (Akiba et al. 2019). This is a framework created to automate and accelerate hyperparameter optimization experiments and continually calls for and assesses the objective function for various parameter values to arrive at the best. In this study, a Tree-Structured Parzen Estimator Sampler (TPESampler) was used to explore the hyperparameter space efficiently. This approach often enables faster identification of optimal hyperparameters compared to grid search, which systematically evaluates all combinations within a predefined grid. Moreover, Optuna supports flexible and complex search spaces, including conditional hyperparameter spaces where the configuration of one hyperparameter depends on the value of another. For each ML algorithm, 100 Bayesian optimization trials were performed to determine the hyperparameters that maximize the AUC. Additionally, the optimization process was enhanced by integrating the Median Pruner to eliminate unpromising trials. The specific details for each ML algorithm, including parameter settings and search space ranges, are listed in Table S3.

To interpret the feature importance of the model, the SHAP (Shapley additive explanations) framework was applied to the models. SHAP provides a model-agnostic approach for interpreting machine learning models by attributing a prediction to the contributions of individual features (Lundberg 2017). It is based on coalitional game theory and Shapley values, providing strong theoretical foundations. It is a local explainability model based on Shapley values. The Shapley value is the average marginal contribution of a feature value across all possible coalitions.

### Model performance evaluation and other prediction tools comparison

To assess the generalizability and superiority of the model performance, three test datasets were used and compared with twenty other prediction tools. The precalculated scores of these tools were obtained from dbNSFP v4.4a (Liu et al. 2020), which includes CADD (Rentzsch et al. 2019), ClinPred (Alirezaie et al. 2018), DEOGEN2 (Raimondi et al. 2017), FATHMM (Shihab et al. 2013), gMVP (Zhang et al. 2022), LIST-S2 (Malhis et al. 2020), M-CAP (Jagadeesh et al. 2016), MetaLR (Dong et al. 2015), MetaRNN (Li et al. 2022), MetaSVM (Dong et al. 2015), MutationAssessor (Reva et al. 2011), MutPred (Li et al. 2009), MVP (Qi et al. 2021), Polyphen2(Hvar) (Adzhubei et al. 2010), PrimateAI (Sundaram et al. 2018), PROVEAN (Choi et al. 2012), REVEL (Ioannidis et al. 2016), SIFT (Ng and Henikoff 2003), VARITY (Wu et al. 2021), VEST4 (Carter et al. 2013). These tools used conservation properties as a foundation for model development and incorporated various combinations of other prediction scores, frequency, functional annotations, structural properties, interactions, domain information, epigenomic features, and other properties as features. CADD, ClinPred, DEOGEN2, M-CAP, MetaLR, MetaRNN, MetaSVM, MutPred, MVP, REVEL, and VARITY incorporated other prediction scores or AF as features, which were known to enhance predictive performance. Tools such as CADD and VEST4, which were designed to predict pathogenic variants in both coding and non-coding regions, also incorporated epigenomic properties. Among the twenty tools, tree-based algorithms such as ClinPred, DEOGEN2, M-CAP, MutPred, REVEL, VEST4, and VARITY were the most commonly used. Additionally, DNN-based algorithms including gMVP, MetaRNN, MVP, and PrimateAI, as well as probabilistic-based algorithms like FATHMM, LIST-S2, MutationAssessor, PROVEAN, and SIFT, were also employed. Furthermore, M-CAP, MetaRNN, MVP, REVEL, VARITY, and gMVP were specifically designed to predict the pathogenicity of rare variants. The thresholds for each prediction tool were based on the dbNSFP or were set as recommended in the original studies.

The eight performance metrics used to evaluate the classification performance of the model included accuracy, precision, sensitivity, specificity, F1-score, Matthews correlation coefficient (MCC), area under the receiver operating characteristic curve (AUC), and area under the precision-recall curve (AUPRC). MCC represents the correlation coefficient between the observed and predicted classifications (Vihinen 2012), and can be measured using the following equation:

$$\:\text{A}\text{c}\text{c}\text{u}\text{r}\text{a}\text{c}\text{y}=\:\frac{\text{T}\text{P}+\text{T}\text{N}}{\text{T}\text{P}\:+\text{T}\text{N}+\text{F}\text{P}+\text{F}\text{N}}$$

$$\:\text{P}\text{r}\text{e}\text{c}\text{i}\text{s}\text{i}\text{o}\text{n}=\:\frac{\text{T}\text{P}}{\text{T}\text{P}+\text{F}\text{P}}$$

$$\:\text{R}\text{e}\text{c}\text{a}\text{l}\text{l}\:(=\text{S}\text{e}\text{n}\text{s}\text{i}\text{t}\text{i}\text{v}\text{i}\text{t}\text{y})=\:\frac{\text{T}\text{P}}{\text{T}\text{P}+\text{F}\text{N}}$$

$$\:\text{S}\text{p}\text{e}\text{c}\text{i}\text{f}\text{i}\text{c}\text{i}\text{t}\text{y}=\:\frac{\text{T}\text{N}}{\text{T}\text{N}+\text{F}\text{P}}$$

$$\:\text{F}1\:\text{s}\text{c}\text{o}\text{r}\text{e}=2\times\:\frac{\text{P}\text{r}\text{e}\text{c}\text{i}\text{s}\text{i}\text{o}\text{n}\:\times\:\text{S}\text{e}\text{n}\text{s}\text{i}\text{t}\text{i}\text{v}\text{i}\text{t}\text{y}}{\text{P}\text{r}\text{e}\text{c}\text{i}\text{s}\text{i}\text{o}\text{n}+\text{S}\text{e}\text{n}\text{s}\text{i}\text{t}\text{i}\text{v}\text{i}\text{t}\text{y}}$$

$$\:\text{M}\text{C}\text{C}=\:\frac{\left(\text{T}\text{P}\times\:\text{T}\text{N}\right)-\left(\text{F}\text{P}\times\:\text{F}\text{N}\right)}{\sqrt{\left(\text{T}\text{P}+\text{F}\text{P}\right)\left(\text{T}\text{P}+\text{F}\text{N}\right)\left(\text{T}\text{N}+\text{F}\text{P}\right)\left(\text{T}\text{N}+\text{F}\text{N}\right)}}$$

where TP, FP, TN, and FN represent true positive, false positive, true negative, and false negative, respectively. The AUC and AUPRC represent the aggregated classification performance across all possible thresholds. The best model was selected based on the highest AUC.

## Results

### Feature analysis

**Fig. 2:**
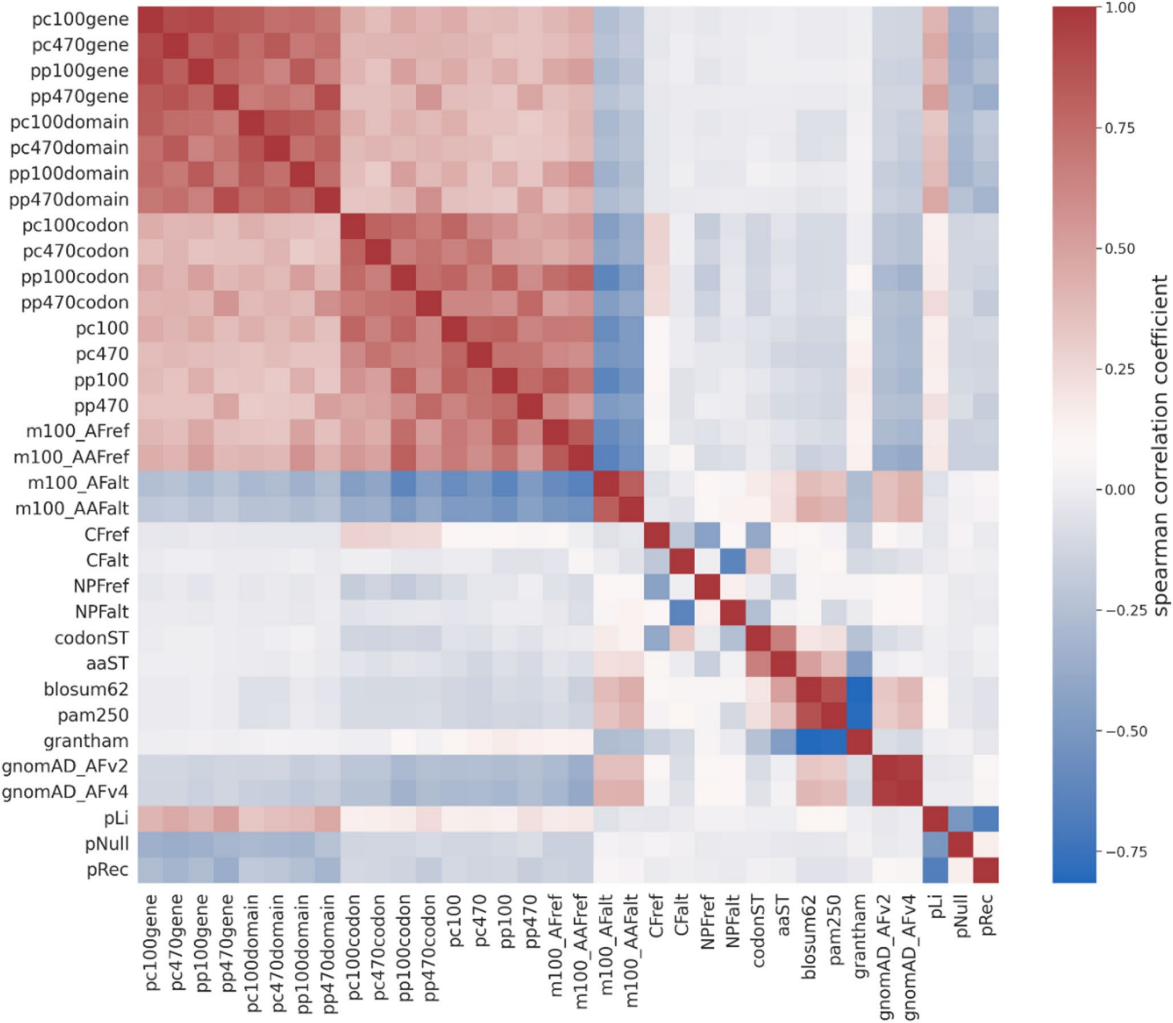
Correlation between 34 features used to train PRP. The heatmap illustrates the Spearman rank correlation coefficients between 34 features for the PRP training dataset

Thirty-four features, classified into four categories, were used to develop the model. Figure 2 illustrates the Spearman rank correlation coefficients calculated among individual features. Most conservation scores show moderate to high positive correlations with each other and with pLi, while showing moderate to weak negative correlations with m100_AAFalt, m100_AFalt, gnomAD_AFv2, gnomAD_AFv4, pNull, and pRec. CFref shows a weak positive correlation with the codon-based conservation score and a moderate negative correlation with codonST and NPFref. Similarly, CFalt shows a weak positive correlation with codonST and a moderate negative correlation with NPFalt. aaST shows a moderate positive correlation with codonST, BLOSUM62 and PAM250, alongside a moderate negative correlation with Grantham. Finally, gnomAD_AF(v2, v4) are highly positively correlated with each other and exhibit a moderate positive correlation with BLOSUM62 and PAM250.

### Model development

Five ML algorithms were applied to identify the most effective model for predicting the pathogenic variants. The best hyperparameters for each ML algorithm, tuned using Optuna, are provided in Table S3. The hyperparameter importance for each model is shown in Fig. S1. The learning rate is the most important hyperparameter for the XGBoost, LightGBM, TabNet, and DNN models, while max_depth is the most important hyperparameter for the CatBoost model.

**Fig. 3:**
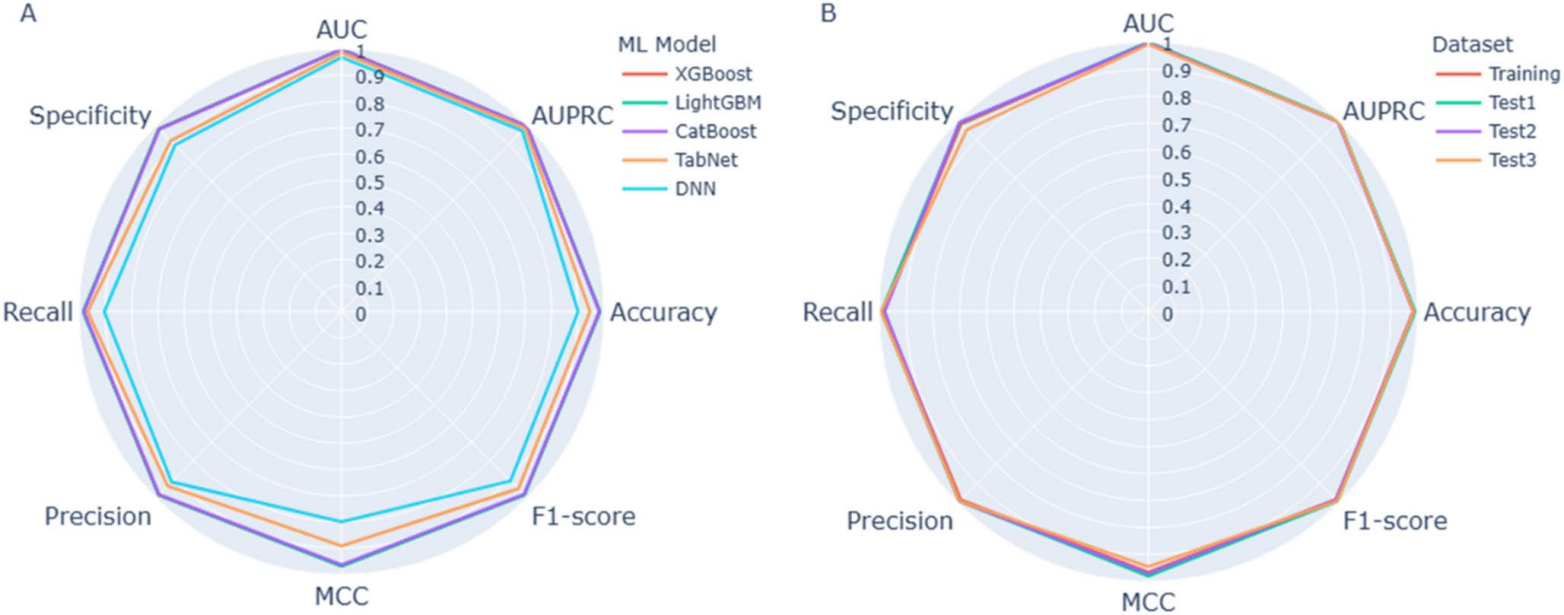
Performance of models in the classification of pathogenic variants. (**A**) The performance of five ML models using the training dataset. (**B**) The performance of three test datasets using the XGBoost model. Each axis represents different performance metrics such as AUC, AUPRC, Accuracy, F1-score, MCC, Precision, Recall, and Specificity. The closer each axis indicates better performance

Figure 3A presents a comparison of the eight performance metrics for each model using a radar plot. The tree-based gradient boosting algorithms XGBoost, LightGBM, and CatBoost exhibit superior performance across all metrics, whereas the DNN performed the worst. TabNet, a deep-learning-based algorithm optimized for structured data, outperformed the DNN but still did not match the performance of the tree-based algorithms. It seems that applying a DNN requires appropriate architecture tailored to genomic data. Among three tree-based algorithms, XGBoost achieved the best performance and was selected as the final model, named PRP. Using 10-fold cross-validation on the training dataset (Fig. S2), PRP achieves the following performance metrics: AUC 0.9983, AUPRC 0.9985, Accuracy 0.9833, F1-score 0.9839, MCC 0.9666, Precision 0.9849, Recall 0.9829, and Specificity 0.9838. The performance metrics for each algorithm are listed in Table S4.

### Performance evaluation and comparison

Three independent test datasets were used to evaluate the generalizability of PRP and compare its performance to twenty previously published pathogenic prediction tools including CADD, ClinPred, DEOGEN2, FATHMM, gMVP, LIST-S2, M-CAP, MetaLR, MetaRNN, MetaSVM, MutationAssessor, MutPred, MVP, Polyphen2(Hvar), PrimateAI, PROVEAN, REVEL, SIFT, VARITY, and VEST4. The performance of PRP on the three test datasets is similar across all eight performance metrics, indicating good generalizability (Fig. 3B). However, the performance of other tools varies depending on the dataset (Fig. S3).

**Fig. 4:**
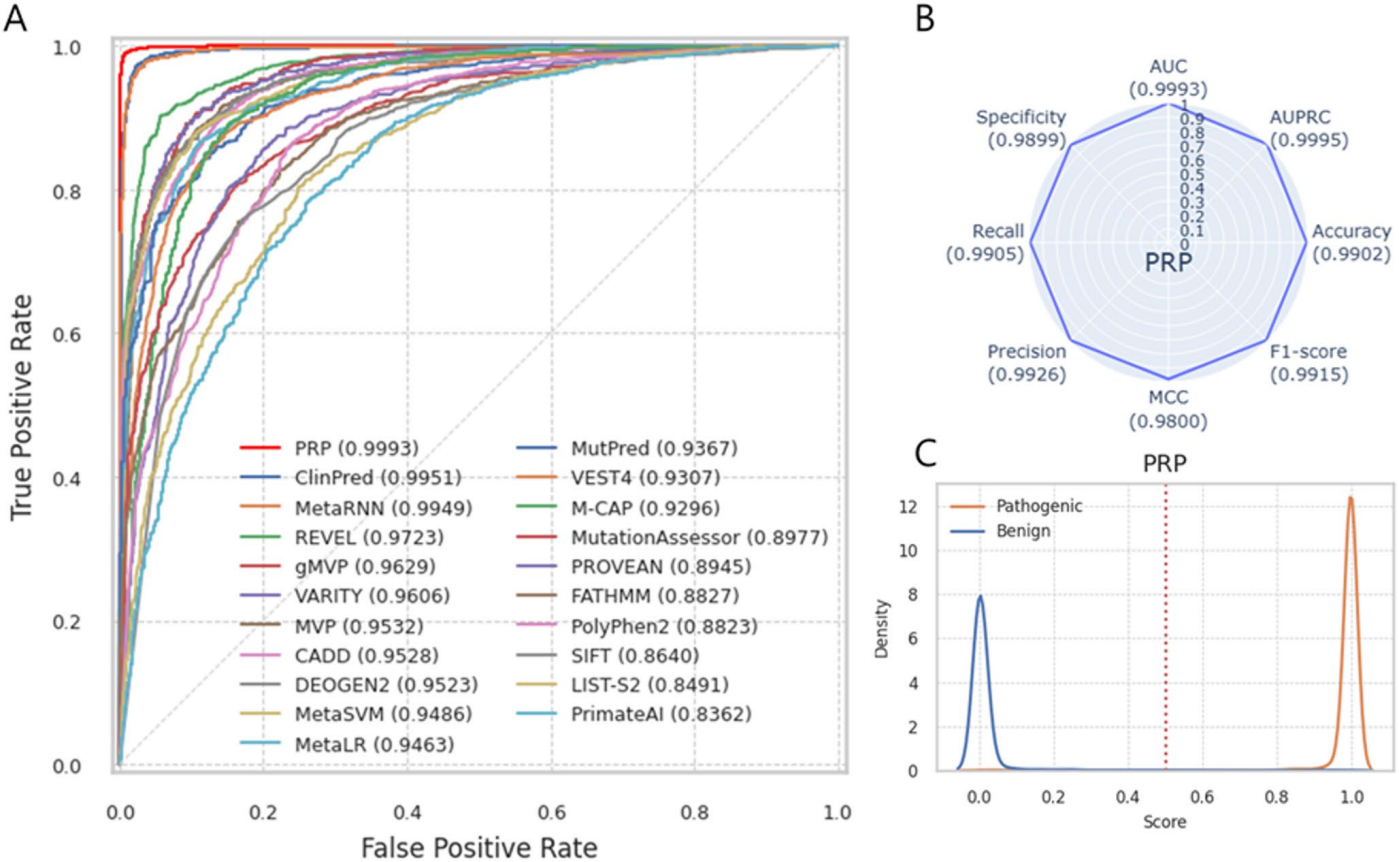
Performance of PRP using test dataset 1. (**A**) ROC curve shows a performance comparison of PRP and 20 other prediction tools. (**B**) Radar Plot shows the eight performance metrics of PRP. Each score indicates in parenthesis. (**C**) The distribution of PRP prediction scores for the pathogenic (orange line) and benign (blue line) variants. The red vertical line indicates the threshold (0.5)

Using Test Dataset 1, which consisted of the latest ClinVar data, including 2,841 TPs and 2,079 TNs, PRP outperforms other prediction tools, achieving the highest AUC of 0.9993 for distinguishing between pathogenic and benign variants, followed by ClinPred (AUC = 0.9951) and MetaRNN (AUC = 0.9949) (Fig. 4A). PRP also demonstrates the best performance across all metrics: AUPRC 0.9995, accuracy 0.9902, F1-score 0.9915, MCC 0.9800, Precision 0.9926, Recall 0.9905, and Specificity 0.9899 (Fig. 4B). The eight performance metrics of the twenty prediction tools are summarized in Table S5 and Fig. S3. The distribution of PRP scores shows a clear bimodal pattern, and using a threshold of 0.5, pathogenic variants can be effectively differentiated from benign variants (Fig. 4C). The distribution of prediction scores from other prediction tools is shown in Fig. S4. The better the performance, the more distinct the distribution of scores, while as performance decreases, there is more overlap between the distributions of pathogenic and benign variants. Test Dataset 1 consists of missense variants (*n* = 3,173, 64.49%), stop_gained variants (*n* = 1,726, 35.08%), start_lost variants (*n* = 18, 0.37%), and stop_lost variants (*n* = 3, 0.06%). Most other prediction tools cover only missense variants, resulting in a missing rate of over 40%, while even CADD, which covers the entire genome, has a missing rate of 7% (Table S5).

**Fig. 5:**
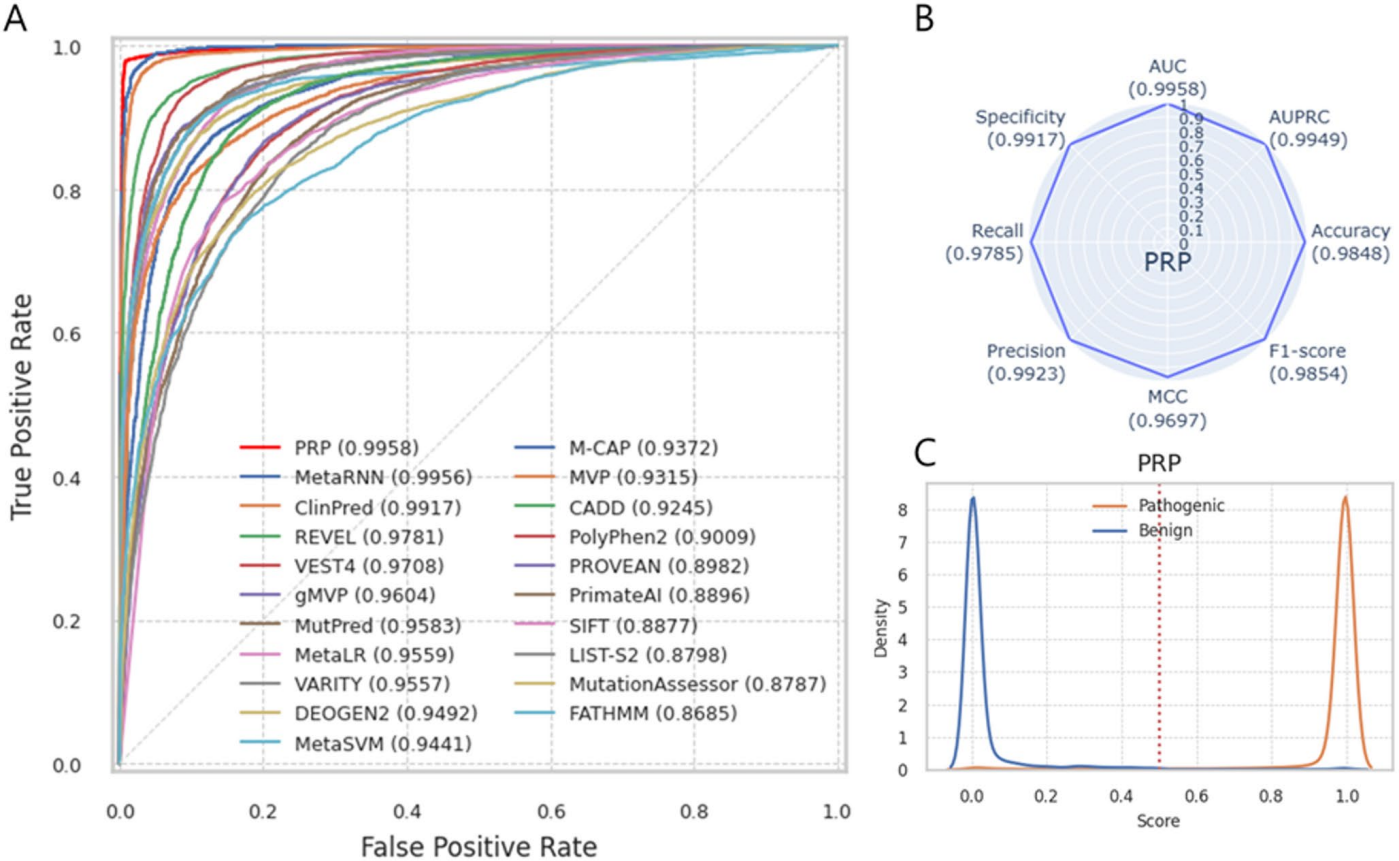
Performance of PRP using test dataset 2. (**A**) ROC curve shows a performance comparison of PRP and 20 other prediction tools. (**B**) Radar Plot shows the eight-performance metrics of PRP. Each score indicates in parenthesis. (**C**) The distribution of PRP prediction scores for the pathogenic (orange line) and benign (blue line) variants. The red vertical line indicates the threshold (0.5)

Test Dataset 2, composed entirely of missense variants from UniProt (*n* = 13,127), demonstrates the best performance of PRP (AUC = 0.9958), surpassing other tools that also used UniProt data for training, including gMVP (AUC = 0.9604), MetaLR (AUC = 0.9559), DEOGEN2 (AUC = 0.9492), MetaSVM (AUC = 0.9441), and MVP (AUC = 0.9315). PRP achieves an AUPRC of 0.9949, Accuracy of 0.9848, F1-score of 0.9854, MCC of 0.9697, Precision of 0.9923, Recall of 0.9785, and Specificity of 0.9917 (Fig. 5A, B, Table S6, Fig. S3). The highest missing rate is observed in MutPred (*n* = 5,880, 44.79%), while MetaRNN and CADD have the lowest missing rate (*n* = 540, 4.11%). PRP prediction scores are highly concentrated around 1 for pathogenic variants, and benign variants are clustered near 0 (Fig. 5C). The distribution of prediction scores from other prediction tools is shown in Fig. S5.

**Fig. 6:**
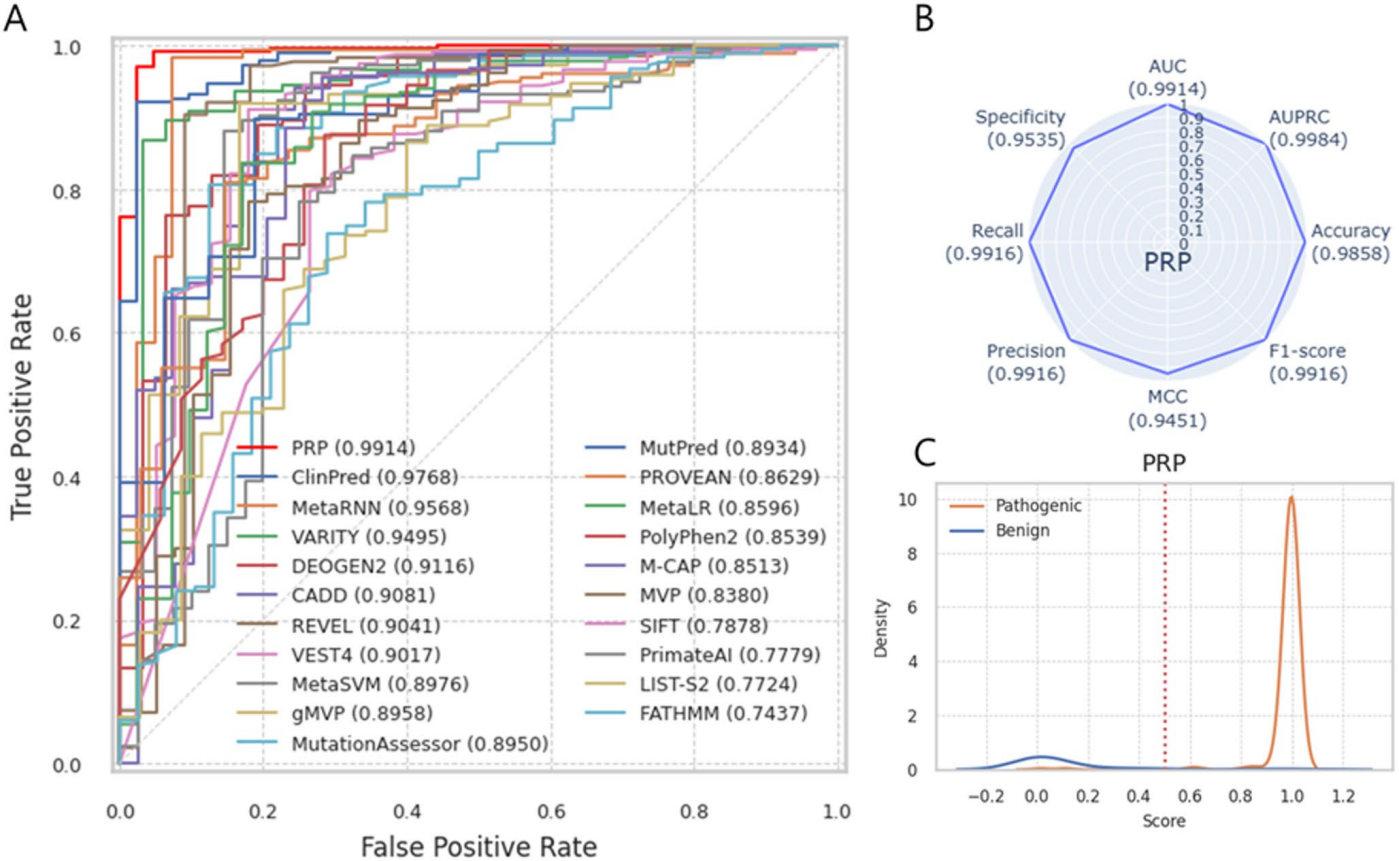
Performance of PRP using test dataset 3. (**A**) ROC curve shows a performance comparison of PRP and 20 other prediction tools. (**B**) Radar Plot shows the eight-performance metrics of PRP. Each score indicates in parenthesis. (**C**) The distribution of PRP prediction scores for the pathogenic (orange line) and benign (blue line) variants. The red vertical line indicates the threshold (0.5)

Using Test Dataset 3, which consists of ClinGen data with 239 TPs and 43 TNs, PRP shows superior performance, achieving an AUC of 0.9914 and AUPRC of 0.9984 (Fig. 6). In contrast, other tools, including ClinPred and MetaRNN, performed poorly on this dataset (Table S7; Fig. S3). The distribution of prediction scores from other prediction tools is shown in Fig. S6. Test Dataset 3 includes missense (*n* = 219, 77.66%), stop_gained (*n* = 54, 19.15%), start_lost (*n* = 8, 2.84%), and stop_lost variants (*n* = 1, 0.35%). The missing rate for most other tools exceeds 20%, with gMVP exhibiting the highest missing rate at 65.25% (*n* = 184) and CADD the lowest at 2.13% (*n* = 6).

### Performance of sensitivity and specificity

**Fig. 7:**
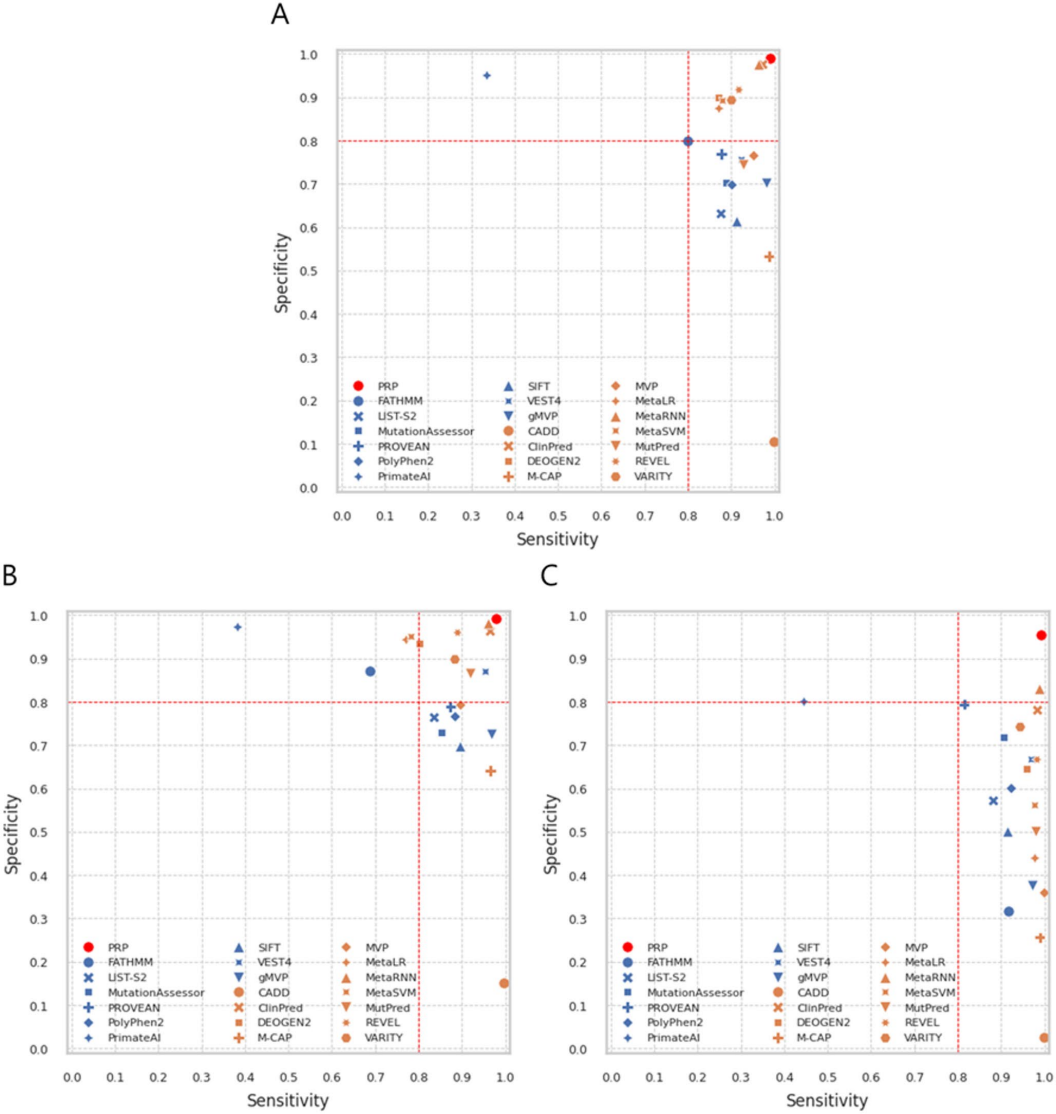
Sensitivity and specificity plot. Three plots illustrate the performance of other prediction tools compared to PRP. Higher sensitivity and specificity indicate better performance. The red marker represents PRP. Ten blue markers represent non-ensemble-based tools, while ten orange markers represent ensemble-based tools, which use other pathogenic prediction scores as a feature. (**A**) Test Dataset (1) (**B**) Test Dataset (2) (**C**) Test Dataset 3

Figure 7 shows the sensitivity and specificity performance of PRP and twenty other prediction tools across three test datasets. The thresholds for each prediction tool were either based on the dbNSFP or set as recommended in the original studies. Consistent with previous research (Li et al. 2018; Niroula and Vihinen 2019), most prediction tools tend to overestimate the number of pathogenic variants, resulting in high sensitivity but low specificity. Except for CADD and M-CAP, most ensemble-based prediction tools that integrate various pathogenic prediction scores as features show less tendency to overestimate sensitivity compared to the non-ensemble-based prediction tools. However, these tools still tend to overestimate the number of pathogenic variants. In contrast, PRP effectively differentiates between pathogenic and benign variants using a threshold value of 0.5, achieving both high sensitivity and high specificity.

### Performance evaluation in rare variants

**Fig. 8:**
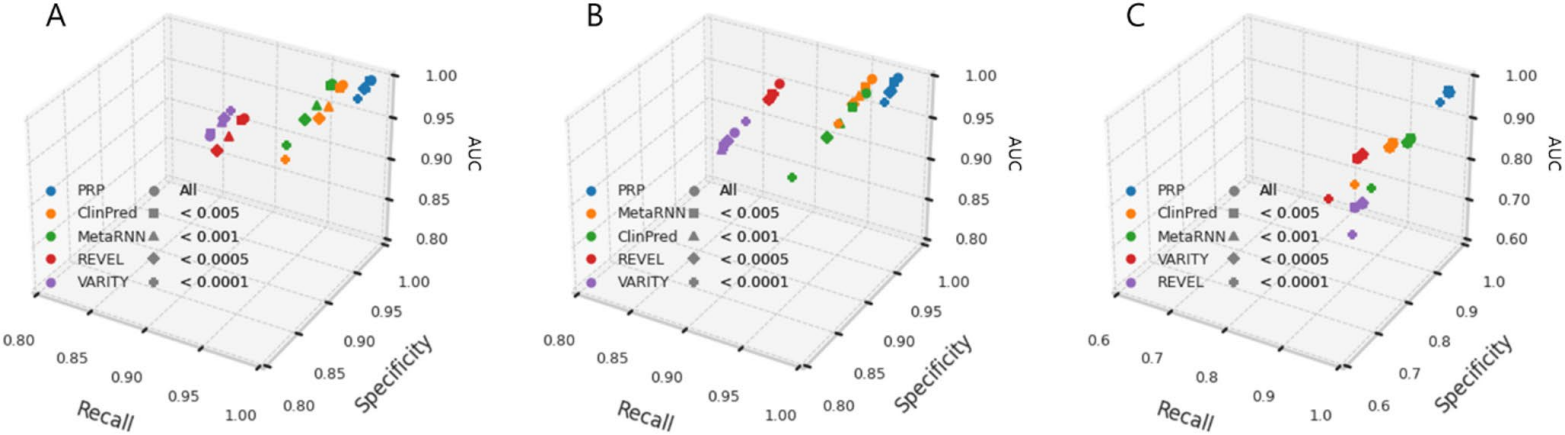
3D scatter plot of AUC, recall and specificity. Three plots illustrate the performance of PRP compared to four other prediction tools across five allele frequency ranges in three test datasets. (**A**) Test Dataset (1) (**B**) Test Dataset (2) (**C**) Test Dataset 3

To assess the performance of PRP in predicting rare variants, its performance was evaluated across five AF ranges and compared with four other prediction tools- ClinPred, MetaRNN, REVEL, and VARITY. ClinPred and MetaRNN incorporated AFs as features, while MetaRNN, REVEL, and VARITY were specifically designed to predict the pathogenicity of rare variants. The performance of PRP remains consistent across all AF ranges, whereas the performance of other tools declines as AF decreases (Fig. 8, Table S8). Specifically, ClinPred and MetaRNN exhibit a notable reduction in specificity, representing the ability to accurately predict true negative (TN) variants, particularly when AF is below 0.001. REVEL, which used data filtered with 0.1% < AF < 1% as the training set, and VARITY, which trained on data with AF < 0.5%, both display consistently low performance across all AF ranges. These results represent the robustness of PRP in distinguishing between pathogenic and benign variants, even when predicting rare variants.

### Feature of importance

**Fig. 9:**
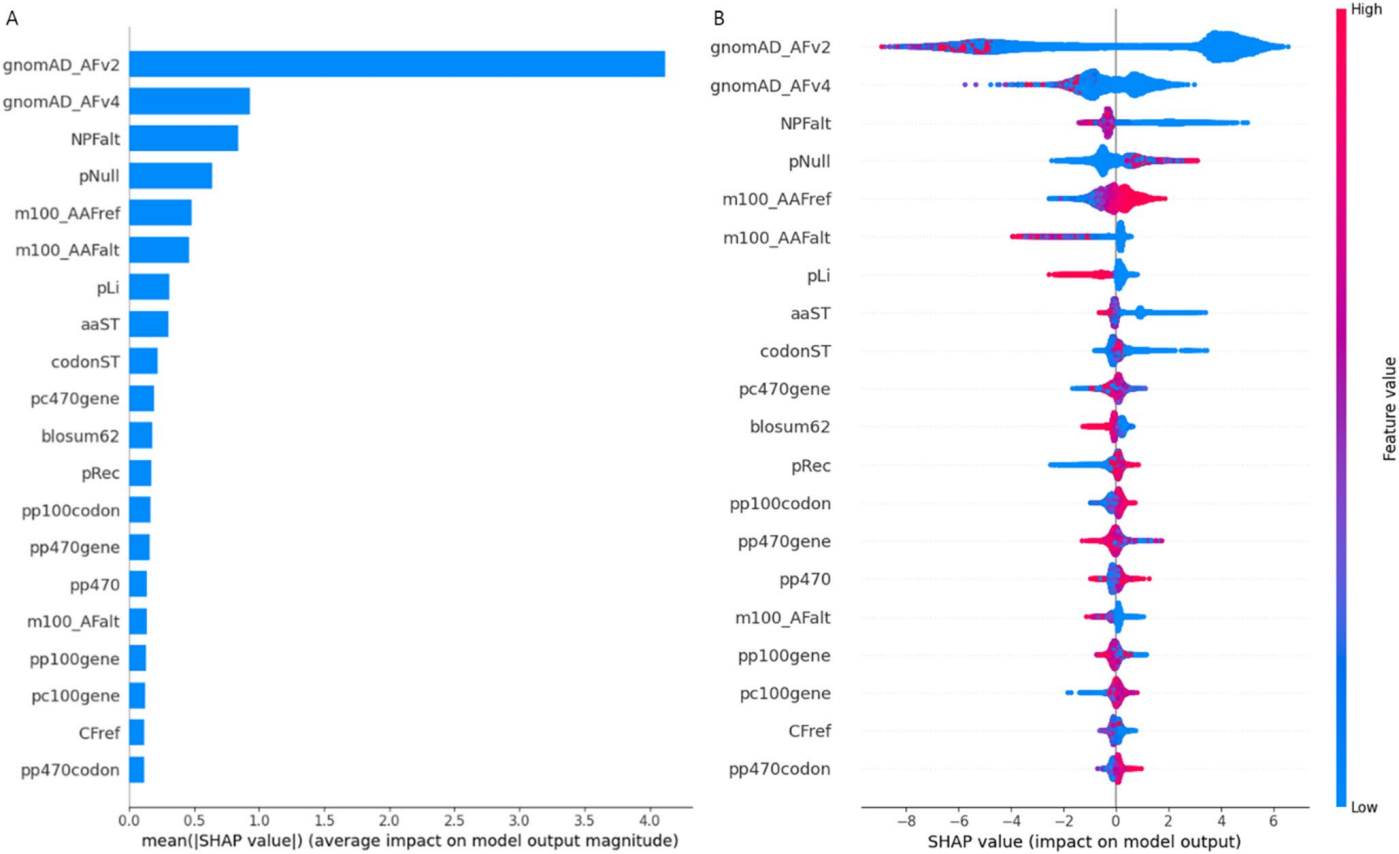
Feature importance for features used in PRP. (**A**) SHAP bar plot shows the most important 20 features for the prediction of pathogenic variants. The x-axis represents each feature’s average absolute SHAP values, and the y-axis displays the features. (**B**) SHAP summary plot combined the top-ranked 20 features importance with feature values. Each dot represents distinct variants color-coded according to the value of corresponding feature on the y-axis and their associated Shapley value on the x-axis. Positive values denote a positive influence on prediction, while negative values suggest a negative influence. The color represents the value of the feature from low to high. Each row represents a feature

SHAP was applied to measure feature importance in the XGBoost model to interpret how features influence the prediction of pathogenic variants. Figure 9 shows the bar and summary plots of the top-ranked 20 features. The summary plot combines feature importance with color-coded feature values. Among the four categories of features, the most important category is frequency, which includes features such as gnomAD_AFv2, gnomAD_AFv4, and NPFalt. gnomAD_AFv2 and gnomAD_AFv4 are the most important features, and the smaller AF results in higher SHAP values, indicating an increased probability of pathogenic variants. In contrast, a larger AF results in lower SHAP values, indicating a decreased probability of pathogenic variants, and thus a higher likelihood of benign variants. This observation is consistent with a previous study(Alirezaie et al. 2018), implying that AF is a crucial feature for predicting pathogenic variants. Lower NPFalt, indicating that amino acids with differing neighbor preferences are less likely to replace each other compared to those with similar neighbor preferences, is associated with a higher probability of pathogenic variants. m100_AAFref and m100_AAFalt are more important than the other conservation scores, such as phasCons and phyloP. Higher m100_AAFref and lower m100_AAFalt are associated with a higher probability of being pathogenic variants. The human-specific substitution metrics, such as aaST and codonST, are found to be more significant than the cross-species substitution metrics like BLOSUM62, PAM250, and Grantham. In all cases, a higher frequency of substitution is corelated with a lower probability of pathogenicity.

**Fig. 10:**
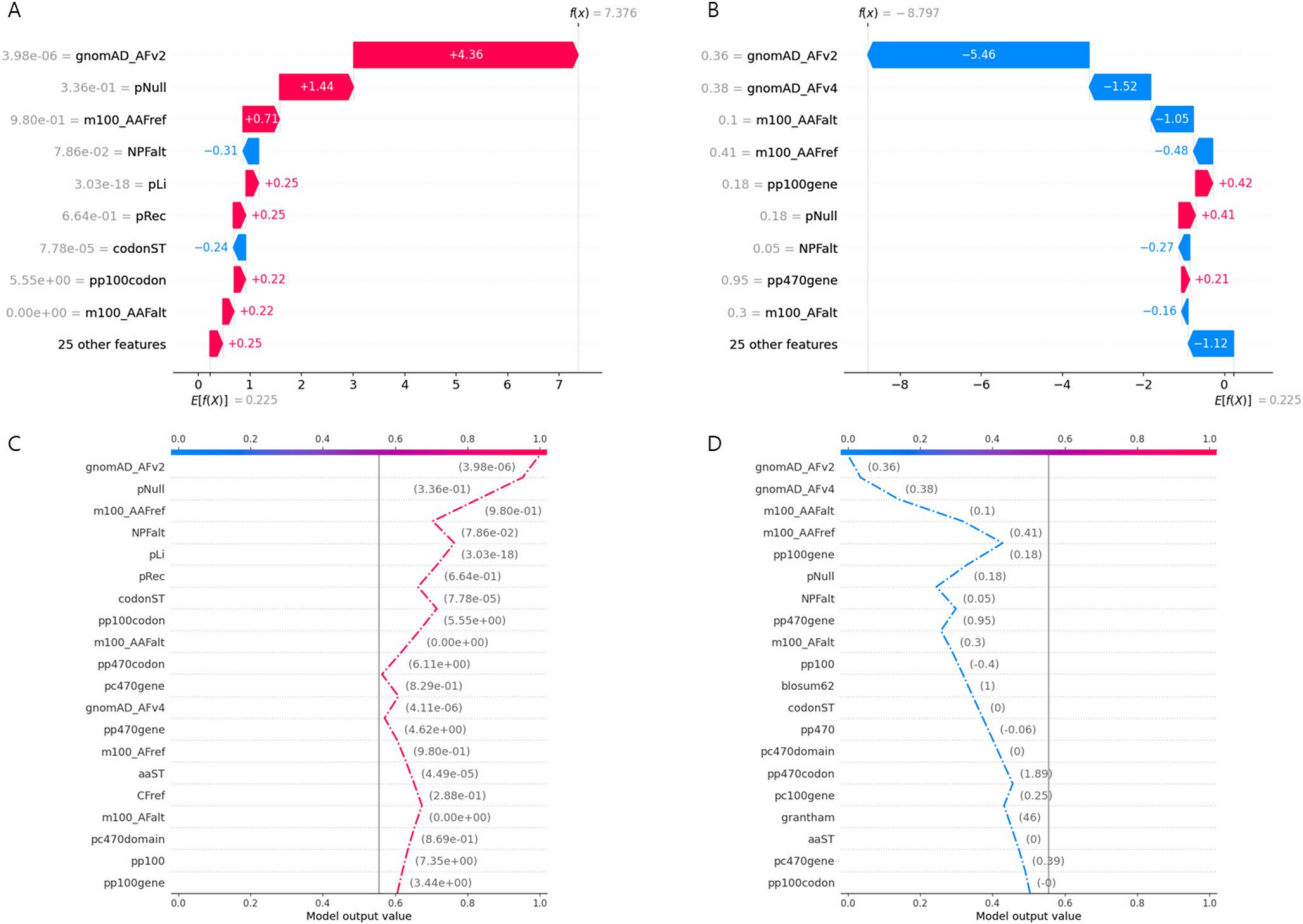
Local interpretation of a single variant. SHAP waterfall plot (**A**) and SHAP decision plot (**C**) of true positive variant. SHAP waterfall plot (**B**) and decision plot (**D**) of true negative variant. In the waterfall plot, red indicates a positive contribution, while blue indicates a negative contribution. The value next to each feature represents the actual value of the feature, and the value next to the color represents the SHAP value. E[f(X)] indicates the expected value of prediction, and f(x) indicates the final prediction in log-odds units. In the decision plot, the x-axis represents SHAP value, converted from log odds to probability and the y-axis displays the features which are ordered by descending importance. The value in parentheses is the actual value of each feature

PRP can interpret variant prediction results using SHAP. Figure 10 shows the waterfall plot and decision plot of the prediction of variants for randomly selected true positive and true negative. These plots allow the interpretation of how each feature contributes to the final prediction.

## Discussion

Distinguishing between pathogenic and benign variants is crucial for the clinical application of genomics. Accurate prediction of pathogenic variants serves as the foundation of personalized medicine, enabling precise diagnosis and individualized treatment through genomic medicine. Despite the availability of several prediction tools, their performance still needs improvement on rare variants.

In this study, PRP improved performance on rare variants and expanded the coverage of variant types compared to other prediction tools by utilizing various novel biological features, without relying on the commonly used prediction scores that others depend on for performance enhancement. PRP leveraged a combination of previously unused features, such as CF, NPF, multiz100way, codonST, and aaST. The PRP method, based on XGBoost - a powerful gradient boosting framework - tuned with Optuna for hyperparameter optimization, and analyzed with SHAP for feature importance, demonstrated superior, generalizable, and interpretable results.

PRP offers several strengths. First, compared to other prediction tools, PRP achieves robust performance across all eight performance metrics on three test datasets. While other tools exhibit varying performance depending on the dataset and tend to overestimate pathogenic variants, leading to imbalances between sensitivity and specificity. PRP consistently achieves high sensitivity and specificity, making it a reliable tool across datasets. Second, PRP improves performance not only for common variants but also for rare variants. Advances in sequencing technology have led to the increasing identification of rare variants, which will constitute a significant proportion of variants of unknown significance. To enhance its ability to discriminate between pathogenic and benign rare variants, PRP includes extremely rare benign variants in its training dataset. PRP consistently demonstrates superior overall performance compared to other tools, particularly in distinguishing pathogenic from uncommon benign variants across a broad range of rare AF thresholds. PRP maintains consistent performance even with rare variants, whereas other prediction tools exhibit significant declines in specificity when handling rare variants. These results indicate the robustness of PRP in accurately distinguishing between pathogenic and benign variants, even under the challenging conditions of rare variant prediction. Third, by using new features without relying on other prediction scores, PRP shows superior performance compared to metaRNN and ClinPred, which depend on multiple prediction scores like SIFT and PolyPheen2. Although incorporating other prediction scores as features has been shown in several studies to enhance discriminative power, this approach risks inflating the performance of tools that rely on external prediction scores. Fourth, PRP covers all types of nsSNVs in the coding region, including missense, start_lost, stop_gained, and stop_lost variants. In contrast, other prediction tools primarily focus on predicting one or a limited set of specific variant types. Most tools specialize in missense variants, while tools like CADD and VEST4 encompass several variant types, including those in both coding and non-coding regions. Tools focusing on missense variants are limited in their ability to handle the diversity of variants present in exome sequencing data, often failing to predict other variant types and resulting in missing values. Furthermore, tools that rely on multiple prediction scores as features are also prone to missing values when one or more of these scores are unavailable.

Among the five ML algorithms tested to determine the most appropriate one for model development, the tree-based gradient boosting algorithms all showed good performance. Tree-based algorithms were the most widely used in pathogenic prediction tools such as ClinPred, DEOGEN2, M-CAP, MutPred, REVEL, VARITY, and VEST4. In addition to PRP, other tree-based tools also outperformed others in performance comparisons. To achieve good performance with a DNN-based algorithm, effectively incorporating genomic information into the architecture appears necessary, as seen in MetaRNN.

Hyperparameter tuning plays a crucial role in maximizing the model performance and generalizability. Grid search is the most common method for optimizing parameters. However, this method lacks a pruning operation, leading to long search times. This study utilized Optuna to efficiently find optimal hyperparameters.

To interpret the contribution of each feature to the prediction of pathogenic variants, PRP utilized SHAP, a tool commonly used to interpret predictions made by ML models. As in previous research(Alirezaie et al. 2018), AFs were identified as the most important features. PRP leveraged AFs from the largest available database, gnomAD, without setting specific thresholds for either pathogenic or benign variants. New features used in PRP, such as NPF, multiz100way, codonST, and aaST, demonstrated greater predictive influence compared to previously utilized features. NPF, which reflects the tendency of amino acids with differing neighbor preferences to replace each other less frequently than those with similar preferences, proves helpful in pathogenic prediction. Additionally, conservation scores at both the DNA and protein levels, derived using different multiple alignment techniques, were incorporated as features. Among these, multiz100way was identified as the most important. Conservation scores based on protein sequence alignment outperformed those based on DNA sequence alignment for pathogenic variant prediction. Human-specific substitution metrics, such as codonST and aaST, were more effective for predicting pathogenic variants than cross-species substitution metrics, such as BLOSUM62 and PAM250. These features are particularly useful for the evaluation of the impact of variants.

While PRP represents a significant advancement, several limitations remain that should be addressed in future studies. Although it incorporates various biological features, it does not integrate structure-based features. Variants can affect the three-dimensional structure of proteins, potentially altering their stability and interaction interfaces. These alterations may disrupt signaling or metabolic pathways, thereby contributing to disease development. Previous tools that incorporate structure-related features have shown limited performance, indicating the need for the identification of more appropriate structure-related features. Furthermore, it is designed to predict the pathogenic risk of single variants. However, since human diseases are often influenced by the combined effects of multiple mutations, considering the potential impact of variant combinations may provide a more comprehensive understanding of disease mechanisms and enhance predictive power. In addition, although it is limited to predicting the effects of variants in coding regions, most of the human genome consists of non-coding sequences. Variants in non-coding regions can also influence disease development by affecting gene regulation, splicing, or chromatin structure. To enhance its utility in clinical genetic diagnostics, PRP needs to be extended to effectively analyze non-coding regions as well. With the increasing discovery of both pathogenic and benign extremely rare variants driven by advances in sequencing technologies, it is essential to identify and incorporate novel biological features that better reflect their distinct characteristics. Additionally, for user convenience, a web interface will be provided, offering access to the PRP scores, biological annotations for variants, and decision plots for prediction interpretation. These efforts will help further improve classification performance and support clinical application.

## Electronic supplementary material

Below is the link to the electronic supplementary material.

Supplementary Material 1

Supplementary Material 2

## Data Availability

The data used in this study are available for download via the link below. ClinGen, https://clinicalgenome.org ClinVar, https://www.ncbi.nlm.nih.gov/clinvar Codon Statistics Database, http://codonstatsdb.unr.edu dbNSFP v4.4a, https://sites.google.com/site/jpopgen/dbNSFP gnomAD, https://gnomad.broadinstitute.org GenBank, https://www.ncbi.nlm.nih.gov/genbank HumsaVar, https://www.uniprot.org/docs/humsavar InterPro, https://www.ebi.ac.uk/interpro/ UCSC, https://genome.ucsc.edu. The training dataset, three test datasets, and codes used to train and test the PRP, as well as to analyze the results, are available at https://github.com/DNAvigation/PRP. The datasets are also accessible via Zenodo at https://doi.org/10.5281/zenodo.15195285.
