# Supplementary material for: PRP: pathogenic risk prediction for rare nonsynonymous single nucleotide variants
Source: Hum Genet. 2025 May 29;144(6):679–94. doi: 10.1007/s00439-025-02751-z (PMC12170803; doi:10.1007/s00439-025-02751-z)
Supplement: Supplementary file 1 — Supplementary Material 1 [file 439_2025_2751_MOESM1_ESM.docx]

**PRP: Pathogenic Risk Prediction for Rare Nonsynonymous Single Nucleotide Variants**

Jee Yeon Heo,^1^ and Ju Han Kim^*,1,2^

^1^Seoul National University Biomedical Informatics (SNUBI), Division of Biomedical Informatics, Seoul National University College of Medicine, Seoul, 03080, Korea

^2^Center for Precision Medicine, Seoul National University Hospital, Seoul, 03080, Korea

^*^Correspondence: juhan@snu.ac.kr


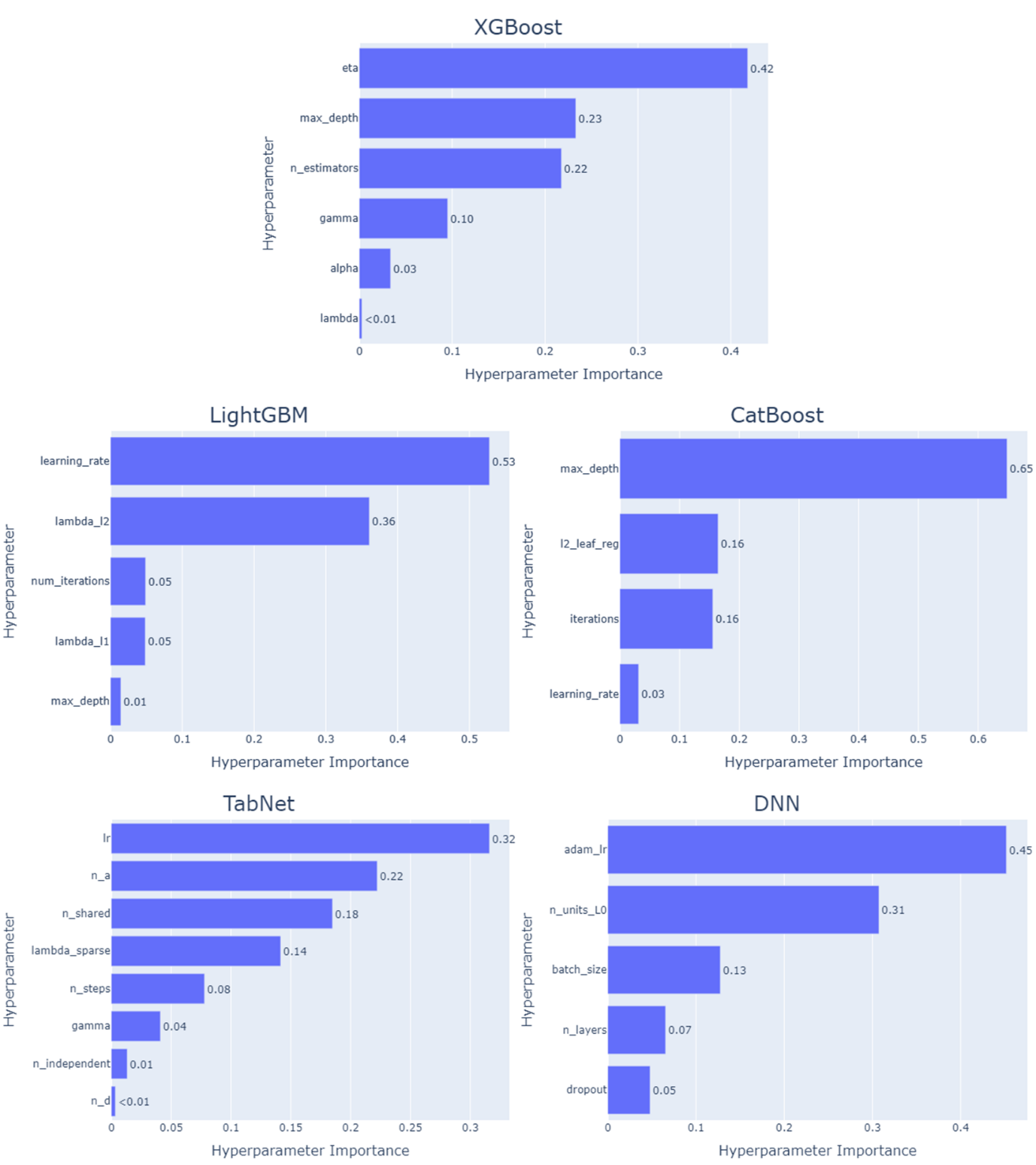


Fig. S1 Hyperparameter importance of five ML algorithms. The eta, lr, adam_lr refer to the learning rate.


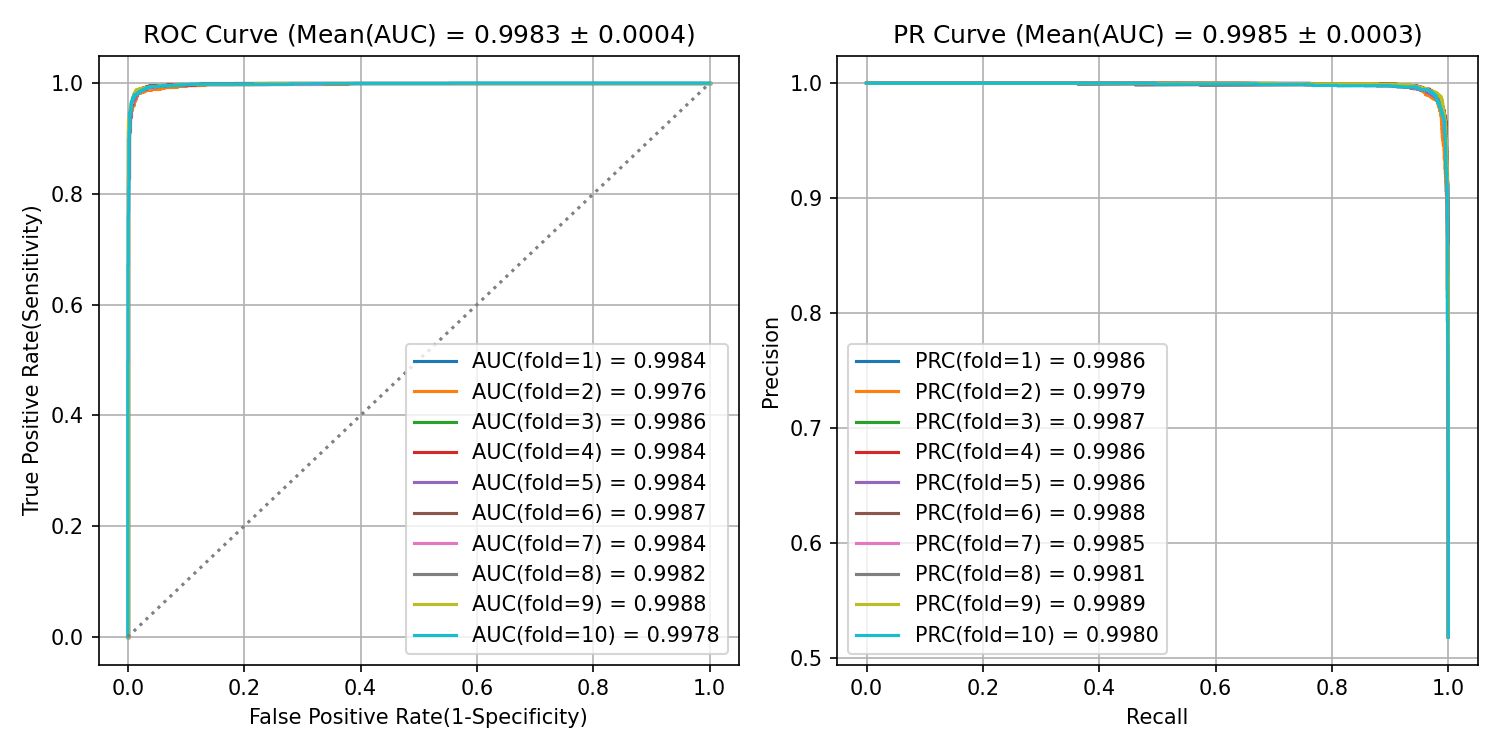


Fig. S2 Receiver Operating Characteristic (ROC) curve and Precision-Recall (PR) curve of XGBoost using 10-fold cross-validation on the training dataset.


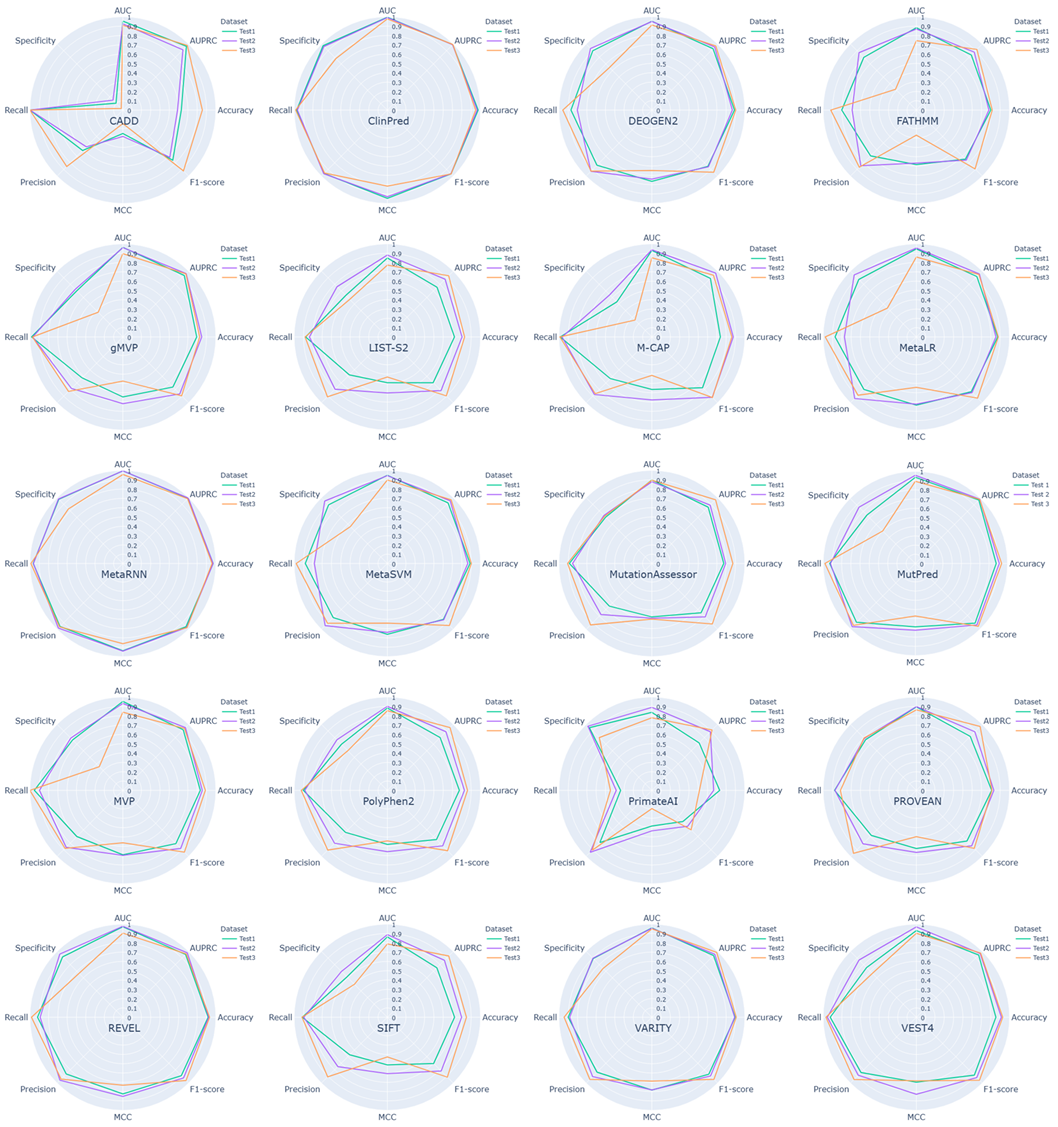


Fig. S3 Performance comparison of the three test datasets across twenty prediction tools. Each axis of the radar plot represents eight performance metrics, AUC, AUPRC, Accuracy, F1-score, MCC, Precision, Recall, Specificity. Closer proximity to the edge of each axis indicates better performance. The green, purple, and orange lines represent Test Dataset 1, Test Dataset 2, and Test Dataset 3, respectively.


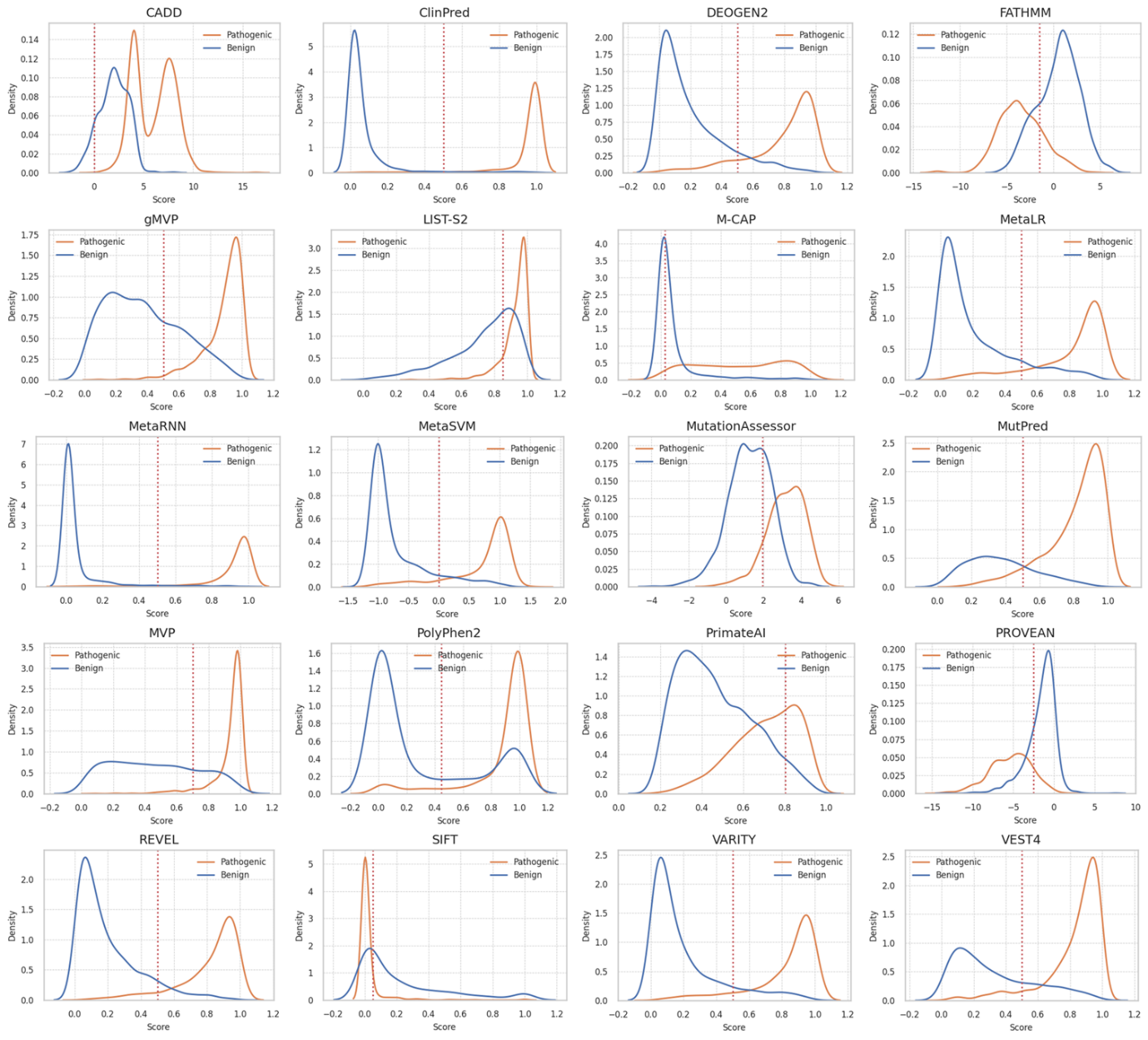


Fig. S4 The distribution of prediction scores from twenty prediction tools in Test Dataset 1 (N=4,920). The orange and blue lines represent pathogenic and benign variants, respectively. The red vertical line indicates the threshold used for classification.


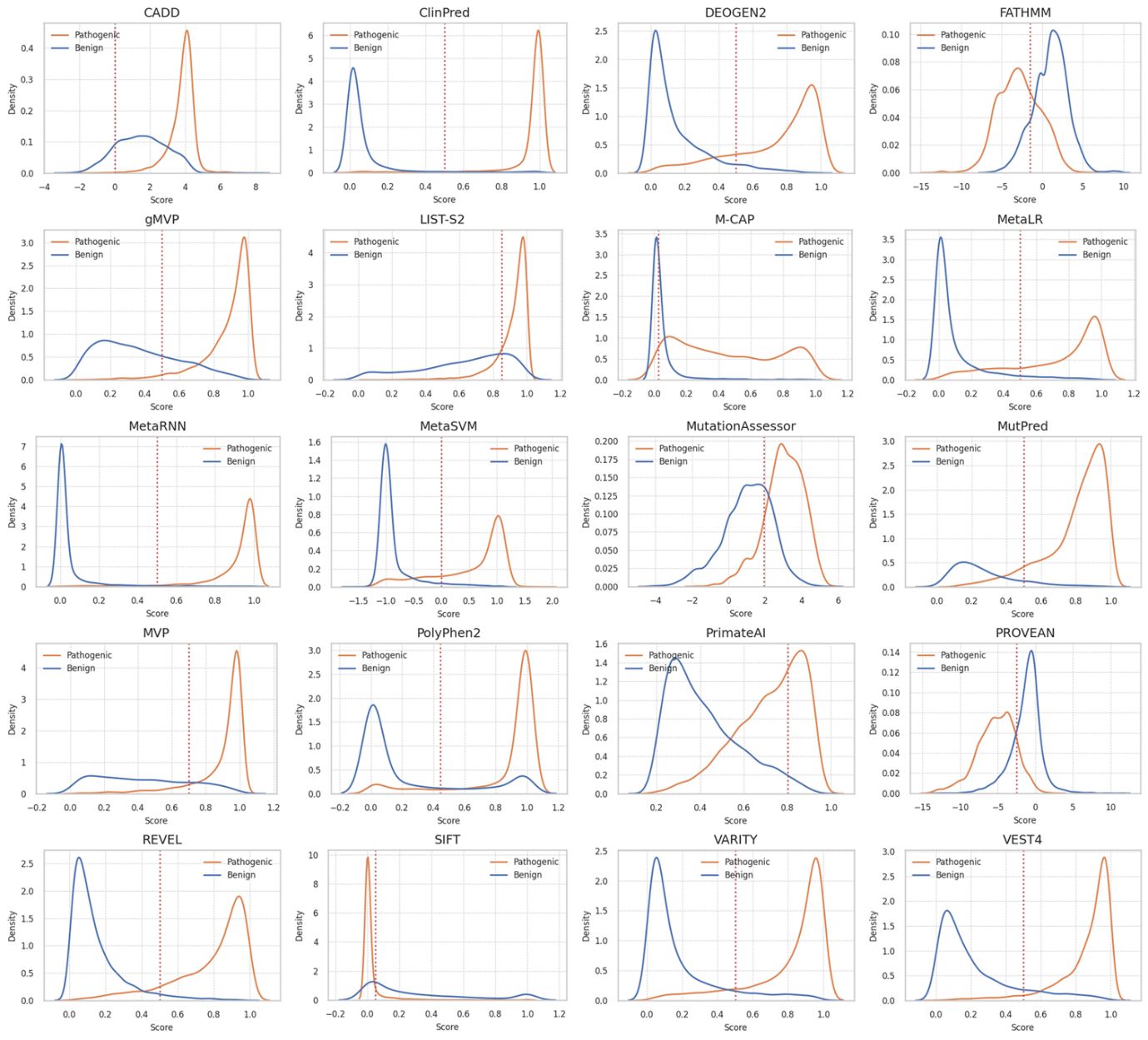


Fig. S5 The distribution of prediction scores from twenty prediction tools in Test Dataset 2 (N=13,127). The orange and blue lines represent pathogenic and benign variants, respectively. The red vertical line indicates the threshold used for classification.


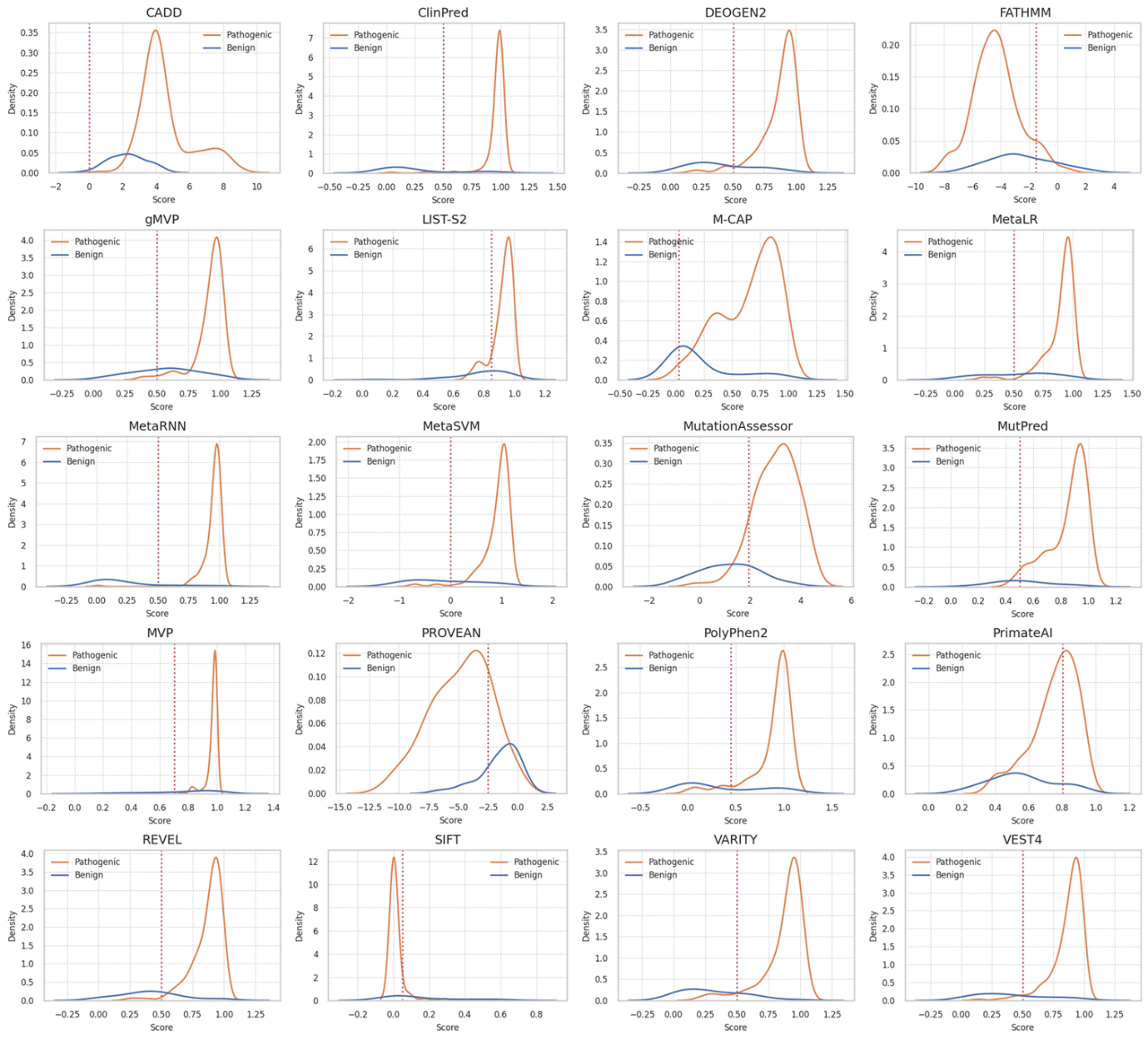


Fig. S6 The distribution of prediction scores from twenty prediction tools in Test Dataset 3 (N=282). The orange and blue lines represent pathogenic and benign variants, respectively. The red vertical line indicates the threshold used for classification.
